# Broad range of research on e-cigarettes

**DOI:** 10.1136/tc-2023-058209

**Published:** 2023-07-19

**Authors:** Joanna E Cohen

**Affiliations:** Institute for Global Tobacco Control, Department of Health, Behavior and Society, Johns Hopkins Bloomberg School of Public Health, Baltimore, Maryland, USA

**Keywords:** Electronic nicotine delivery devices, Advertising and Promotion, Public policy

This issue brings together a collection of papers about e-cigarettes using a range of study designs and data sources, in a range of geographic locations and addressing a range of policy domains.

Eleven papers address marketing. Shah *et al* find that Instagram is a global unregulated marketplace for electronic nicotine delivery systems (ENDS), with little age verification for flavoured products.^1^ Vassey *et al* also focus on Instagram, and report that in 2020 there was an interconnected network of e-cigarette influencers worldwide, with most not restricting youth access to their promotional content.^2^ An assessment of Instagram in 2018 focusing on JUUL posts, by Kostygina *et al*, finds that the vast majority used recruitment or trial-based appeals including smoking cessation and product sampling and programmes, and almost three-quarters used reinforcement or addiction-related appeals such as loyalty programmes.^3^ Braznell *et al* asked people from 22 countries to engage in live webchat communications on the IQOS website, which they conclude can be another opportunity for companies to make claims about their products that may not be warranted.^4^

Keeping with the marketing theme but moving to country-specific studies, Blackwell *et al* conducted an experiment with UK youth aged 13–17, finding that among those who visit retail stores regularly (and who passed the study’s attention checks), seeing e-cigarette retail displays was associated with increased susceptibility to smoking and decreased harm perceptions of smoking.^5^ An Industry Watch by Boston *et al* reports on an unintended consequence of a New Zealand law whereby specialist ‘stores-within-a-store’ emerged to sell e-cigarettes; we have come to learn well that the tobacco industry exploits any loopholes to skirt the spirit of a law, but papers such as this one allow jurisdictions to learn from others and build upon their experiences.^6^ In another Industry Watch, Mus *et al* describe a troubling access channel they observed in Guatemala City—vending machines selling disposable e-cigarettes with no age verification.^7^ A survey of owners and managers of retail stores selling IQOS and HEETS in Israel by Bar-Zeev *et al* finds that Philip Morris International is using similar marketing tactics as they have used for cigarettes, including direct promotional activities.^8^

From the USA, an Industry Watch by O’Leary *et al* recounts a success story in thwarting an industry marketing initiative. The Cheyenne River Sioux Tribe used its sovereign power to redirect JUUL from achieving a proposed ‘partnership’.^9^ Henderson *et al* report on the high prevalence of IQOS marketing within retail stores in Atlanta.^10^ Majmundar *et al* used Google search queries and sales data in the USA, finding that JUUL’s removal of their sweet and fruit-flavoured products resulted in increases in Puff Bar search queries and sales until the House passed a flavoured e-cigarette ban bill; after a steep decline in Puff Bar sales when they suspended sales, the decline slowed after Puff Bar relaunched their products as containing ‘synthetic nicotine’.^11^ These findings remind us that squeezing one part of the policy balloon usually results in an expansion of the balloon elsewhere.

This issue also contains papers addressing policy domains other than marketing. Cotti *et al* describe how they generated a public database of US state and local standardised e-cigarette tax data.^12^ Shang *et al* report on ENDS excise tax incidence for US jurisdictions and examine how tax incidence varies by whether a specific or ad valorem tax base is used, finding that the relationship is moderated by product type (eg, open systems); they discuss the trade-offs between the different types of taxes, along with the added complications of the wide range of design features across the ENDS market.^13^ A discrete choice experiment conducted by Buckell *et al* among US adults who smoke but have a low interest in quitting found that neither a proposed menthol ban nor cigarette taxes encouraged much self-reported intention to switch from cigarettes to e-cigarettes.^14^ Chen *et al* present an analysis of the US Population Assessment of Tobacco and Health survey data from 2017 to 2019 which suggested that those using e-cigarettes to help with their quit attempt were less likely to quit than those using no quit aid or a pharmaceutical aid, though the authors point out that the high-nicotine e-cigarettes that are popular today were not used by many participants at the time of their analysis.^15^

An experimental online survey among 18–25 year-olds in Columbia by Gantiva *et al* found that health warning labels on e-cigarette liquids attracted less attention than other parts of the liquid vial and did not increase perceptions of addictiveness, indicating that more work needs to be conducted to develop appropriate and effective health warning labels for e-cigarettes.^16^ Measey *et al* report on a public opinion survey in Australia where parents supported restrictions on the use of e-cigarettes.^17^ In a study of US state tobacco control spending, Tauras *et al* find that prevalence and intensity of e-cigarette use among high school students was lower in states that spent more on tobacco control.^18^ Boynton *et al* conducted an online survey of US adolescents to evaluate a range of e-cigarette prevention ads and make recommendations for messages that seem to be more effective for preventing youth use of e-cigarettes.^19^

This issue contains two papers related to e-cigarette, or vaping product, use-associated lung injury (EVALI), a serious medical condition that damaged the lungs of people who had used vaping products. Liber *et al* used sales scanner data, finding a decrease in e-cigarette sales in response to news about EVALI and state policies banning the sale of some or all e-cigarettes; cigarette sales were mostly unaffected, however, except in Massachusetts where sales of cigarettes had a temporary increase, primarily of brands used by young people.^20^ A survey of US adults who smoke, by Wackowski *et al*, indicates that after a year following the peak in EVALI deaths, there remained misperceptions about the cause of the outbreak and half of respondents indicated that EVALI reduced their interest in using e-cigarettes in the future.^21^

There are additional contributions included in this issue that increase our understanding of e-cigarettes. Gu *et al* conducted a survey of low-income adults in the US state of California, finding that using e-cigarettes was a risk factor for food insecurity and that using both e-cigarettes and cigarettes was associated with greater risk of food insecurity than using cigarettes alone.^22^ Hourani *et al* computed nicotine flux—the rate of nicotine emission per second—for a range of tobacco products; they report a wide range of nicotine flux across products, that the upper limit of nicotine flux has been increasing for ENDS since 2015, and suggest that nicotine flux could be a useful regulatory target.^23^ Last but not least, Fenton *et al* evaluate the ethical arguments put forward to maximise ENDS’ potential to reduce harm, conclude that the reasoning in these arguments is flawed and recommend further assessment of ENDS from an ethical perspective to help inform policy.^24^

The papers in this issue provide further insight into e-cigarettes and can inform what might be done to maximise their benefits and reduce their harms. Clearly, the heterogeneity of the e-cigarette—for example, the range of design features of the devices and liquids that influence their appeal, the variation in use patterns and the extent to which they deliver nicotine and other chemicals to users—poses many challenges for research as well as for regulation. The continued rapid change in the products that are available and how they are marketed does not help. From a policy perspective, we need to remain open-minded about the interventions that could achieve our policy goals, evaluate their impacts and unintended consequences and remember that policies that address a very circumscribed target may result in a ‘whack-a-mole’ state where our ‘solution’ yields another problem popping up elsewhere.
